# Volcanic ash ice nucleation activity is variably reduced by aging in water and sulfuric acid: the effects of leaching, dissolution, and precipitation†

**DOI:** 10.1039/d1ea00071c

**Published:** 2021-12-22

**Authors:** William D. Fahy, Elena C. Maters, Rona Giese Miranda, Michael P. Adams, Leif G. Jahn, Ryan C. Sullivan, Benjamin J. Murray

**Affiliations:** Center for Atmospheric Particle Studies, Carnegie Mellon University Pittsburgh, Pennsylvania 15213 USA; School of Earth and Environment, University of Leeds Leeds LS2 9JT UK; Department of Chemistry, University of Cambridge Cambridge CB2 1EW UK ecm63@cam.ac.uk; Faculty of Geosciences, Geoengineering, and Mining, Technische Universität Bergakademie Freiberg 09599 Freiberg Germany

## Abstract

Volcanic ash nucleates ice when immersed in supercooled water droplets, giving it the potential to influence weather and climate from local to global scales. This ice nucleation activity (INA) is likely derived from a subset of the crystalline mineral phases in the ash. The INA of other mineral-based dusts can change when exposed to various gaseous and aqueous chemical species, many of which also interact with volcanic ash in the eruption plume and atmosphere. However, the effects of aqueous chemical aging on the INA of volcanic ash have not been explored. We show that the INA of two mineralogically distinct ash samples from Fuego and Astroni volcanoes is variably reduced following immersion in water or aqueous sulfuric acid for minutes to days. Aging in water decreases the INA of both ash samples by up to two orders of magnitude, possibly due to a reduction in surface crystallinity and cation availability accompanying leaching. Aging in sulfuric acid leads to minimal loss of INA for Fuego ash, which is proposed to reflect a quasi-equilibrium between leaching that removes ice-active sites and dissolution that reveals or creates new sites on the pyroxene phases present. Conversely, exposure to sulfuric acid reduces the INA of Astroni ash by one to two orders of magnitude, potentially through selective dissolution of ice-active sites associated with surface microtextures on some K-feldspar phases. Analysis of dissolved element concentrations in the aged ash leachates shows supersaturation of certain mineral species which could have precipitated and altered the INA of the ash. These results highlight the key role that leaching, dissolution, and precipitation likely play in the aqueous aging of volcanic ash with respect to its INA. Finally, we discuss the implications for understanding the nature and reactivity of ice-active sites on volcanic ash and its role in influencing cloud properties in the atmosphere.

## Abstract 2

The ice nucleation activity of volcanic ash can change when exposed to liquid water or aqueous sulfuric acid through a complex interplay of geochemical alteration mechanisms.

## Graphical abstract

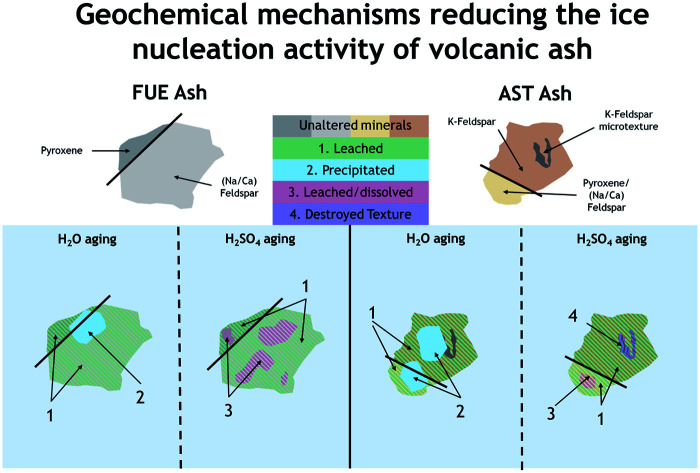

**Environmental significance**

Volcanic ash nucleates ice in cloud droplets, influencing cloud properties and their role in climate. However, its ice nucleation activity (INA) may change when exposed to atmospheric aging processes, limiting understanding of the potentially evolving impact of ash on clouds as it disperses away from the volcano. Here we exposed two volcanic ash samples to water or sulfuric acid on atmospherically relevant time scales and observed a variable reduction of INA over time, likely through different mechanisms of chemical alteration acting on ice-active pyroxene and feldspar minerals. These mechanisms could limit the formation of ice in volcanically influenced clouds and be applied to future work studying the alteration of other mineralogical ice-nucleating materials common in the atmosphere.

## 1 Introduction

Volcanic ash from explosive eruptions can serve as ice-nucleating particles (INPs) in the atmosphere and may be especially important far from mineral dust sources of INPs such as arid- and semi-arid regions.^1–4^ While global annual mean emissions of volcanic ash (176–256 Tg a^−1^)^5^ are an order of magnitude lower than those of mineral dust (1000–2000 Tg a^−1^),^6^ ash loadings in the atmosphere regularly meet or exceed mineral dust loadings on regional scales, and can even surpass the global annual mean dust emissions during a single large event (*e.g.*, >5000 Tg released during the 1991 Pinatubo eruption, Philippines).^7^ Eruptions of various magnitudes can inject volcanic ash into the upper troposphere and lower stratosphere, and while coarse grains (>63 μm) rapidly sediment to the ground, smaller super- and submicron particles have been reported to remain airborne for up to 6 months following an eruption and spread 1000 s of kilometers from the source volcano.^8,9^

Volcanic ash is a silicate-based material consisting of glass and crystalline components, which can include feldspar, pyroxene, olivine, biotite, amphibole, mica, quartz and iron(–titanium) oxide minerals,^10^ depending on the source magma composition and storage and ascent conditions.^11^ All natural ash samples tested in the laboratory to date have acted as INPs when immersed in supercooled water.^12–18^ The presence and properties of crystalline components in the ash are thought to exert a strong influence on its ice nucleation activity (INA), while the glass component has been shown to nucleate ice relatively poorly.^16^ Several primary minerals have been implicated as the ice-active phases in volcanic ash in previous studies, including alkali (Na/K-rich) feldspar, plagioclase (Na/Ca-rich) feldspar, and pyroxene.^14–17^ Similarly, K-feldspar (hereafter used to refer to K-rich alkali feldspar), Na/Ca-feldspar, and quartz are recognized as ice-active phases in mineral dust,^19–27^ while secondary minerals in dust such as kaolinite, illite and montmorillonite may also nucleate ice.^20,24,28–32^ Ice nucleation on mineral surfaces is thought to occur at specific nanoscale sites^33–36^ due to some combination of chemical and physical features, for instance relating to crystal structure, microscale texture and resulting geometry, and the presence and orientation of chemical functionalities such as hydroxyl groups.^37–42^

In addition to the mineralogy of the ash bulk, the history of a given ash sample following eruption is likely to influence its INA, potentially by modifying the properties of the ash surface. For example, the INA of ash might be enhanced or depressed by interaction with volcanic gases (*e.g.*, H_2_O, SO_2_, HCl) at high temperatures in the eruption plume, with the effect influenced by the susceptibility of individual minerals in the ash to thermochemical alteration.^43^ As airborne ash emerges from the hot eruption plume core and begins cooling and dispersing with the entrainment of air, ash particles are likely to develop a liquid film and/or become immersed in liquid droplets.^44,45^ The liquid phase can be acidified by uptake of acids (*e.g.*, SO_2_/H_2_SO_4_, HCl, HF, HNO_3_) released during the eruption and/or encountered during atmospheric transport.^46–49^ Studies on mineral dusts and their crystalline components have shown that such ‘chemical aging’ by exposure to water,^25,50^ aqueous acids,^50–52^ or acid vapor^53–55^ can sometimes reduce the INA of these materials. A recent study found the INA of Fuego volcanic ash to be unaltered or reduced by aqueous chemical aging.^56^ The variable effects on INA observed are strongly INP type- and aging method-dependent, and their underlying mechanisms are poorly understood, in part due to limited knowledge of how ice-active surfaces are altered by exposure to different species. While mechanistic understanding of the chemical aging of volcanic ash at ambient temperature is lacking in the context of atmospheric ice nucleation, insights into the geochemical reaction mechanisms involved may be gained from existing literature on aqueous alteration of silicate minerals commonly found in ash.

For many multi-oxide silicate minerals (*e.g.*, feldspar, pyroxene, amphibole, mica) immersed in near-neutral or low pH aqueous solution, alteration begins with rapid leaching of metal cations (*e.g.*, Na^+^, K^+^, Ca^2+^, Mg^2+^, Fe^2+^) from interstices of the silicate network by exchange with protons (H^+^/H_3_O^+^) in solution.^57–61^ This can produce a surface ‘leached layer’ depleted in metal cations and enriched in H^+^/H_3_O^+^, silicon (Si), and aluminum (Al) that is in substitution of Si in the silicate network tetrahedra. Alternatively or additionally, an amorphous, hydrated, cation-depleted and Si(/Al)-enriched layer can form through a dissolution–reprecipitation process at the mineral–solution interface.^62–66^ The precise nature and formation mechanisms of leached and/or reprecipitated layers on silicate surfaces are still debated (*e.g.*, see reviews by Chardon *et al.*, Wilson, Yuan *et al.*)^67–69^ but this discussion is beyond the scope of our study.

**Fig. 1:**
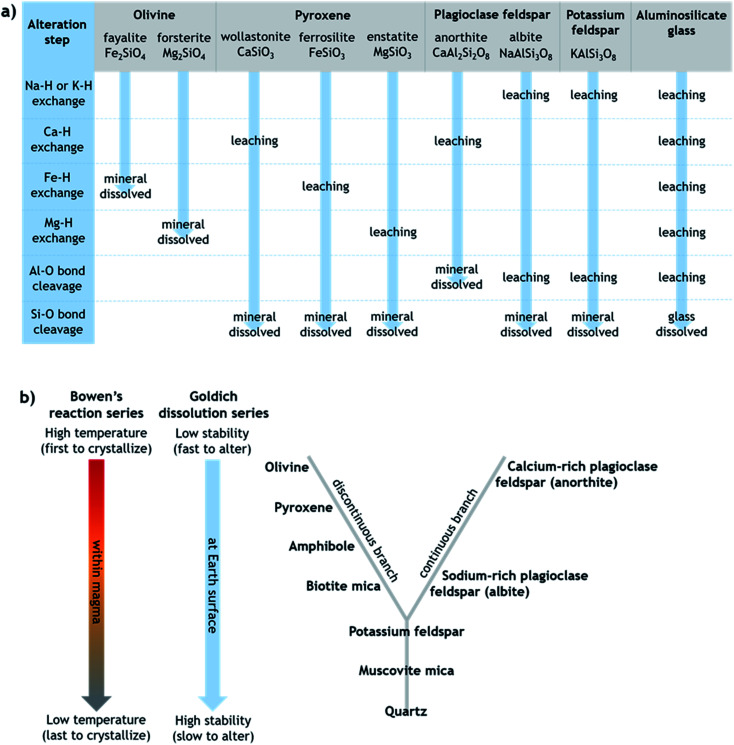
(a) Simplified schematic of the alteration steps of various silicates that can be present in volcanic ash, modified from Oelkers.^61^ Note that olivine dissolves directly by metal–proton exchange since silica tetrahedra occur as single units, whereas other silicates dissolve by Si–O (or Al–O in anorthite) bond cleavage between connected tetrahedra. (b) Schematic depicting the relative order of silicate minerals in terms of their crystallization from cooling magma (Bowen's reaction series) and their stability against alteration under Earth surface conditions (Goldich dissolution series), modified from Huddart and Stott.^73^ The unifying idea is that minerals that crystallized from magma at temperatures farthest from those at the Earth's surface are most susceptible to alteration when exposed at the Earth's surface.

Generally, the extent of surface alteration depends on various factors including the mineral composition, the pH of the aqueous solution, the cation and anion concentrations in the solution, and the mineral–solution contact duration.^70^ Further, the growth of any leached layer may become limited by the rate of cation/proton diffusion through it and by dissolution of the silicate network itself at the mineral surface.^57^ While the mechanisms involved vary across minerals in ways that are still not fully understood, Fig. 1a provides a simplified summary proposing key steps of leaching and dissolution of several silicates, where in most cases the final step is cleavage of Si–O bonds between network tetrahedra by H^+^/H_3_O^+^.^61^ All else being equal, the relative rates of alteration of silicate minerals can broadly be considered in relation to their (in)stability at Earth surface conditions, as outlined in the Goldich dissolution series, which follows Bowen's reaction series, in Fig. 1b.^71–73^ Accordingly, minerals such as olivine, pyroxene and Ca-rich feldspar, which crystallize from cooling magma at higher temperatures (earlier), are expected to alter more readily in aqueous solution at ambient temperature compared to minerals such as K-feldspar and quartz, which crystallize from cooling magma at lower temperatures (later).

Considering this knowledge from the literature, depending on the mineralogical make-up of volcanic ash and the conditions of chemical aging encountered during atmospheric transport, leaching and dissolution will likely occur in parallel and at different rates on ash surfaces, and may influence the INA of the ash. To gain insight specifically into how volcanic ash interaction with an aqueous droplet at near-neutral or low pH affects its INA, here we conduct experiments suspending two mineralogically contrasting ash samples in water (H_2_O_(l)_) or sulfuric acid solution (H_2_SO_4(aq)_) over different atmospherically relevant time scales, prior to ice nucleation measurements. To explore how changes in INA between ‘non-aged’ and ‘aged’ samples might relate to alteration of specific mineral phases in the ash, dissolved element concentrations are analyzed in the aging solution from each experiment and compared with INA data using correlation coefficients and a learned predictive model between the two measurements. We then present a series of hypotheses on how combinations of geochemical alteration processes might explain complex trends in INA observed during aging of the ash in H_2_O or H_2_SO_4_, before proposing implications and further research to better constrain the potential impacts of volcanic ash on atmospheric ice nucleation.

## 2 Materials and methods

### 2.1 Volcanic ash samples

Ash samples from the Fuego (FUE) and Astroni (AST) volcanoes were chosen for study (Table 1) based on their contrasting composition and INA, as reported previously by Jahn *et al.* and Maters *et al.*^14,16^ The INA of the more crystalline basaltic–andesitic FUE ash is likely driven by pyroxene, while that of the more glassy trachyphonolitic AST ash is probably dominated by K-feldspar. Each specimen was mechanically milled prior to chemical aging experiments (see Section 2.2) using a zirconia ceramic mill ball and jar to produce atmospherically relevant particles sizes. Milling also exposed fresh surfaces for the purpose of testing the effect of chemical aging on INA under controlled conditions, independent of any influence on INA of prior alteration of ash surfaces while airborne, once deposited, or during storage. The Brunauer, Emmett, and Teller specific surface area (SSA_BET_) of each milled sample (Table S1†), and of samples collected following chemical aging (Table S2†), was determined after overnight degassing from a ten-point N_2_ adsorption isotherm at −196 °C using a Micromeritics TriStar 3000 instrument.^74^

**Table 1:** Properties of the volcanic ash samples studied

Ash sample (code)	Source eruption	Classification^c^	Mineralogy^d^ (wt%)							
			Glass	Alk. Feld.	Plag. Feld.	Ortho pyrox.	Clino pyrox.	Oliv.	Crystal. SiO_2_	Fe (–Ti) oxide
Fuego ash (FUE)^a^	Volcán de Fuego, Guatemala, February 2015	Basaltic–andesite	20	3	68	3	4	2	m. c	m. c.
Astroni ash (AST)^b^	Astroni volcano, Italy, 3.8–4.4 ka	Trachy-phonolite	72	19	7	—	2	—	—	m. c.

a Mineralogy reported in Jahn *et al*.^14^

b Mineralogy reported in Maters *et al*.^16^

c Based on bulk elemental composition (Table S1) according to the total alkali *versus* SiO_2_ classification scheme for igneous rocks.^75^

d Alk. feld. = Na/K-feldspar, Plag. feld. = Na/Ca-feldspar, orthopyrox. = orthopyroxene (compositions between Mg_2_Si_2_O_6_ and Fe_2_Si_2_O_6_ solid solution, with small amounts of Ca^2+^ substitution possible), clinopyrox. = clinopyroxene (other compositions of solid immiscibility, particularly with higher Ca^2+^ content), oliv. = olivine, crystal. SiO_2_ = crystalline SiO_2_, m. c. = minor component below ∼2 wt% quantification limit by X-ray diffraction.

### 2.2 Chemical aging experiments

Batch experiments of FUE or AST ash suspended in MilliQ H_2_O (18.2 MΩ cm) or pH 1.75 ± 0.5 (0.01 M) H_2_SO_4_ (93% VWR Normatom trace metal grade) at room temperature, at an ash-to-solution ratio of 1 : 100 by mass, and under constant gentle rotation at 1 rpm were carried out to explore the effects of ash–liquid interactions on ash INA (see Section 2.3). This ash-to-solution ratio falls within the wide range of estimated particle concentrations (10^−4^ to >10^3^ g L^−1^) in liquid aerosols and cloud droplets.^76^ Aqueous aging was chosen to allow time-resolved chemical analysis of dissolved material, providing insight into the geochemistry occurring at the ash–liquid interface and in the aging solution. Moreover, this approach enables longer aging time scales to be studied compared to typical chamber or flow tube reactor experiments. As in previous atmospheric processing studies of ash,^77,78^ H_2_SO_4_ was selected as it is readily formed by oxidation of volcanic SO_2_ and is likely the dominant acid species to interact with ash in the atmosphere.^79^ While mixtures of species would more accurately mimic atmospheric conditions, these simpler experimental conditions facilitate isolation of key alteration mechanisms that may modify ash INA. Experiments were run in triplicate for different durations (minute to day scale) and then filtered by vacuum through a pre-rinsed 0.45 μm polyethersulfone membrane filter to achieve a desired ash–liquid contact time of 10 min, 1 h, 4 h, 24 h, or 120 h. The collected ash was dried in air for 24 h on a weighing boat beneath a plastic cover, and then stored at room temperature in a sealed plastic vial in a desiccator until ice nucleation measurements and subsequent SSA_BET_ analysis. The collected liquid was filtered further by syringe through a pre-rinsed 0.2 μm polyethersulfone membrane filter and acidified to ∼1.5 vol% HNO_3_ (67% Fisher Chemical trace metal grade) for storage at ∼4 °C prior to dissolved Si, Al, Fe, Mg, Ca, Na, K, Ti, and Mn analysis by inductively coupled plasma optical emission spectroscopy (ICP-OES; Thermo Fisher iCAP 7400 Radial). The corresponding limits of detection and quantification (LOD and LOQ) are given in Table S2.† Blank experiments with pure MilliQ H_2_O or pH 1.75 ± 0.5 H_2_SO_4_ (containing no added ash) were also run for 10 min, 1 h, 4 h, 24 h, or 120 h, and then filtered, acidified, and stored as above prior to analysis by ICP-OES.

### 2.3 Ice nucleation measurements

The INA of the non-aged and aged ash samples was determined using the Microlitre Nucleation by Immersed Particles Instrument (μL-NIPI) described in detail by Whale *et al.*^80^ This instrument has previously been used to characterize a variety of ice-active materials relevant to this study including volcanic tephra and glass,^15,16,43^ K- and Na/Ca-feldspars,^21,81^ and other silicate minerals.^25,82^ For each non-aged and aged ash sample, a separate 1, 0.2, and 0.04 wt% suspension in MilliQ H_2_O was made gravimetrically and was vortexed to ensure an even distribution of particles. These three suspension concentrations were chosen to widen the temperature range over which to measure INA and observe potential aging effects. For each suspension, an array of 30 to 40 1 μL droplets was pipetted onto a hydrophobic silanized glass cover slip placed on a temperature-controlled stage (Grant-Asymptote EF600 Stirling Cryocooler) and cooled from room temperature to 0 °C at −5 °C min^−1^. The droplets were then exposed to a temperature ramp of −1 °C min^−1^ until all droplets were frozen, with freezing recorded by a digital camera for subsequent analysis. Uncertainty in temperature is estimated to be ±0.4 °C.^80^ A 0.2 L min^−1^ N_2(g)_ flow over the droplets prevented condensation and frost formation. Background freezing spectra of pure MilliQ H_2_O (containing no added ash) were taken each day to characterize the INA of impurities in the H_2_O and interactions between droplets and the cover slip.^83^ Freezing spectra are reported as the ice nucleation active site density normalized to mass (*n*_m_) or surface area (*n*_s_) as a function of temperature (*T*) following eqn 1 to facilitate comparisons of INA within this study and with other experimental setups.^84,85^1
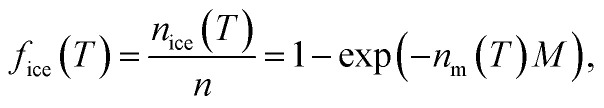
where *f*_ice_(*T*) is the fraction of droplets frozen and *n*_ice_(*T*) is the cumulative number of droplets frozen at *T*, *n* is the total number of droplets in the experiment, and *M* is the mass of solid sample suspended per droplet. To calculate the surface area normalized freezing spectrum, *M* would be replaced by *A*, the total surface area of the sample per droplet as calculated from *M* and SSA_BET_. Uncertainties in the freezing spectra were calculated as discussed by Vali^86^ by modeling each experiment as a collection of Poisson processes (see the ESI for further details†).

## 3 Results

### 3.1 Ice nucleation activities of non-aged and aged ash samples

**Fig. 2:**
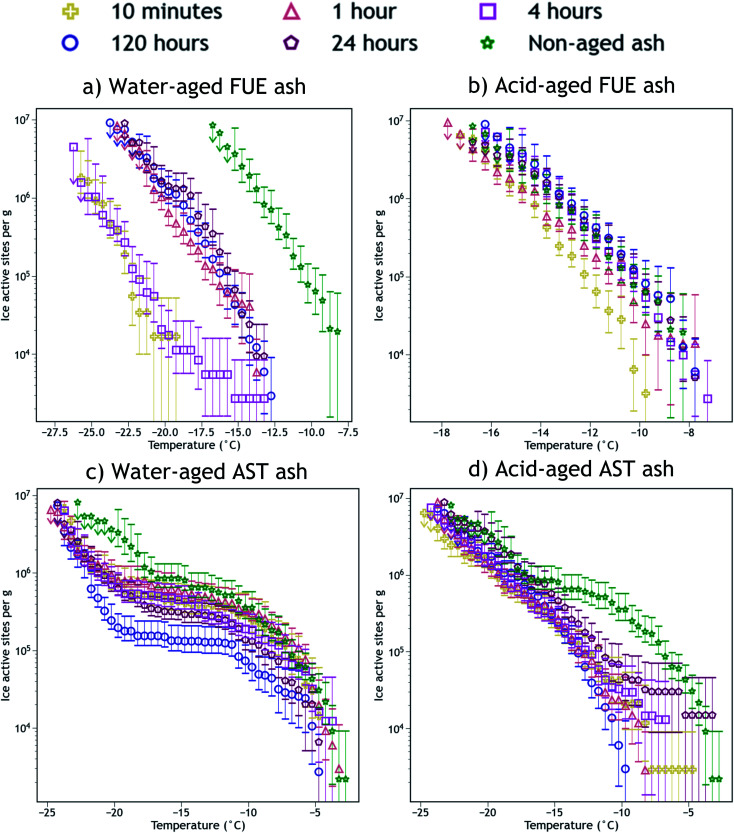
Ice nucleation active site density normalized to mass (*n*_m_) *versus* temperature spectra of FUE ash non-aged or aged for different durations in (a) H_2_O (water-aged) or (b) pH 1.75 H_2_SO_4_ (acid-aged), and AST ash non-aged or aged for different durations in (c) H_2_O (water-aged) or (d) pH 1.75 H_2_SO_4_ (acid-aged). The 95% confidence intervals were approximated as the 2.5^th^ and 97.5^th^ quantiles using Monte Carlo simulations based on a Poisson distribution of droplet freezing events. The background of each spectrum (a freezing experiment with pure water droplets measured the same day) has been subtracted and the data from the 1, 0.2, and 0.04 wt% suspension experiments have been averaged into a single spectrum. Further details of error analysis and background subtraction are presented in the ESI.†

The INA of the non-aged and aged FUE and AST ash samples is reported in Fig. 2. Aging in H_2_O or H_2_SO_4_ always reduced the INA of the ash, but the magnitude of this reduction appears to be sensitive to the specific ash, aging solution type, aging duration, and immersion freezing temperature being considered. The change in INA associated with each aging experiment is summarized in Tables S2 and S3.†

Exposure of FUE ash to H_2_O caused a reduction in INA of either 5 or 10 °C (a 10 or 1000× decrease in *n*_m_ at any given temperature). The magnitude of this decrease is non-monotonic with respect to time, as after 10 min and 4 h in H_2_O the aging effect is markedly stronger (10 °C) than after 1, 24, and 120 h (5 °C). In contrast, exposure of FUE ash to H_2_SO_4_ caused only a slight reduction in INA of 1 to 2 °C (a ∼5× decrease in *n*_m_ at any given temperature) after 10 min of aging, followed by a recovery of INA such that aging times of 1 to 120 h did not significantly reduce freezing temperatures relative to the non-aged sample.

Exposure of AST ash to H_2_O appeared to have a time-dependent effect on INA above −17 °C. After 10 min to 1 h of aging, little to no deactivation is observed, while after 4 h of aging, a borderline insignificant loss is observed. After 24 h, this reduction in INA becomes significant, and after 120 h a maximum reduction in *n*_m_ by a factor of 5 to 10 occurs above −10 °C, persisting to −17 °C because relatively few sites become active between −10 °C and −17 °C. Below −17 °C, the early aging effects are different, as after 10 min in H_2_O, freezing temperatures are reduced by 3 to 4 °C (a ∼5× decrease in *n*_m_). This level of INA is not further altered until 120 h of aging in H_2_O, where the decrease in *n*_m_ reaches a factor of 10 near −17 °C, although at the coldest temperatures (<−22 °C) this additional reduction in INA is not observed.

Again contrasting with the effects of H_2_O, exposure of AST ash to H_2_SO_4_ caused most of the ice nucleation sites active above −17 °C to be immediately and irreversibly lost from the freezing spectrum. While the freezing spectrum of the ash aged for 24 h does overlap with that of the non-aged ash at the warmest temperatures (>−8 °C), note the large error bars which indicate that there is not a statistically significant recovery of INA at these temperatures after aging in H_2_SO_4_. Only a few of these ice-active sites above −8 °C appear to remain from 10 min to 24 h of aging, represented by a switch from a negative concavity of the non-aged AST ash spectrum to a flat or slightly positive concavity of the aged ash spectrum. The portion of the spectrum below −17 °C remains essentially unaltered at all time points, similar to the observed stabilization of INA in FUE ash exposed to H_2_SO_4_.

The SSA_BET_ measurements of the ash samples (Tables S1 and S2†) enable detection of changes in their surface area due to chemical aging. Little change in SSA_BET_ is observed for FUE and AST ash samples exposed to H_2_O, except for a slight increase for AST ash aged for 120 h, whereas SSA_BET_ is raised by a factor of five for both ash samples exposed to H_2_SO_4_, meaning there is more ash surface area per unit mass post-aging. Since ice nucleation is localized to the ash surface, this change could impact the trends observed in INA in Fig. 2. To deconvolute surface area changes from other aging effects on INA, we also present surface area normalized ice nucleation active site density (*n*_s_) freezing spectra as a function of temperature in Fig. S1.† In the *n*_s_ spectra of FUE ash, the slightly lower INA observed upon exposure to H_2_SO_4_ for 10 minutes relative to all later time points becomes less significant, while the overall loss of INA from non-aged to acid-aged FUE and AST (below −17 °C) becomes statistically significant. The other changes in INA presented in Fig. 2 are not altered significantly depending on the normalization scheme. Since *n*_m_ reflects the aggregate of all aging effects that could influence volcanic ash while *n*_s_ removes the effects of changing surface area, *n*_m_ will be used for the remainder of this study, however, changes to SSA_BET_ as a result of the aging process will be discussed where appropriate.

### 3.2 Dissolved element concentrations in chemical aging solutions

Mean dissolved element signatures from the aging experiments are reported in Table S2† as the ratio of their molar abundance relative to Si in the aging solution (as measured by ICP-OES) over their molar abundance relative to Si in the non-aged bulk ash sample (as measured by XRF). A value for this metric above unity (*i.e.*, X/Si *s*/*b* > 1, where X is a metal element, s refers to the aging solution, and *b* refers to the bulk ash) is presumed to reflect preferential release of X from the ash surface (*e.g.*, by leaching/dissolution) and/or preferential removal of Si from the aging solution (*e.g.*, by precipitation). The Si concentration in the aging solution normalized to the measured SSA_BET_ is also reported as an indicator of overall dissolution of the ash. Silicon was chosen as a reference element as it is typically the last to be released from the silicate network and is thus often used as a proxy for silicate dissolution.^61^

The major trends in dissolved element signatures are generally consistent with the reaction steps shown in Fig. 1. That is, we infer fast leaching of elements that occur in interstices of the silicate network such as alkali and alkaline earth metals (Na, K, Ca, and Mg), followed by slower dissolution of elements such as Al and Si that exist in tetrahedra of the silicate network. However, without knowledge of the chemical composition and spatial distribution of glass and crystalline components at the ash surface as well as their individual rates of leaching and dissolution, the contributions of different phases in the ash to the dissolved element signatures observed are difficult to disentangle.

## 4 Discussion

The mechanisms by which chemical aging in aqueous solution impairs or reduces the INA of volcanic ash have not been previously investigated or reported in the literature. However, based on the available geochemical data (*e.g.*, ash mineralogy, leachate chemistry), we present hypotheses to explain the observed ice nucleation phenomena. Variations in the effect depending on the specific ash sample, aging solution type, and aging duration are inferred to reflect parallel competing changes in INA of the ash during leaching and dissolution of its component phases and precipitation of secondary phases. In agreement with conclusions from previous studies,^14,16^ the INA of FUE ash is likely driven by pyroxene while that of AST ash is probably dominated by K-feldspar above −17 °C and pyroxene or Na/Ca-feldspar below −17 °C.

### 4.1 Analysis of correlations between ice nucleation spectra and dissolved element signatures

To quantify the complex correlations between changes in INA (see Section 3.1, Tables S3 and S4†) and mean dissolved element signatures (see Section 3.2, Table S2†) of the aged ash samples, a vector representing a numerical approximation of the difference in INA of ash at each aging duration compared to the INA of the non-aged ash was calculated by projecting each cumulative freezing spectrum onto a 10^th^-order Chebyshev polynomial basis set.^87^ The INA difference vector was then determined by calculating the quotient of the polynomial coefficients of the aged ash and non-aged ash freezing spectra. Element-wise Pearson correlation matrices of all dissolved element signatures and vector coefficients for all experiments divided by ash/aging solution pair are reproduced in Fig. S2–S5† in the ESI.†^88^ Correlations and anticorrelations will be referred to as ‘strong’ (>0.67), ‘weak’ (between 0.67 and 0.33), or ‘no correlation’ (<0.33).

For FUE ash aged in H_2_O, dissolved Ca, Na, and Mg concentrations are variably but positively correlated with one another (Fig. S2†), consistent with their release in particular by leaching of Na/Ca-feldspar, Mg-rich pyroxene, and/or olivine (as in Fig. 1; see Section 4.2). However, for FUE ash exposed to H_2_SO_4_, dissolved Mg is not correlated with other alkali and alkaline earth metals, instead having a weak relationship with Si (Fig. S3†), potentially reflecting leaching and dissolution of Mg-rich pyroxene and/or olivine (see Section 4.4). For AST ash aged in H_2_O or H_2_SO_4_, dissolved Ca, Na, and K are always strongly correlated (Fig. S4 and S5†), and are probably released readily by leaching of glass, feldspar, and pyroxene components of the ash surface. Note that correlations between ice nucleation difference descriptors (the coefficients of the divided Chebyshev polynomials) and dissolved element signatures indicate that the release of elements from the ash correlates positively with changes in INA, while the reverse is true for anticorrelations. Specific examples will be highlighted where relevant in the following sections.

**Fig. 3:**
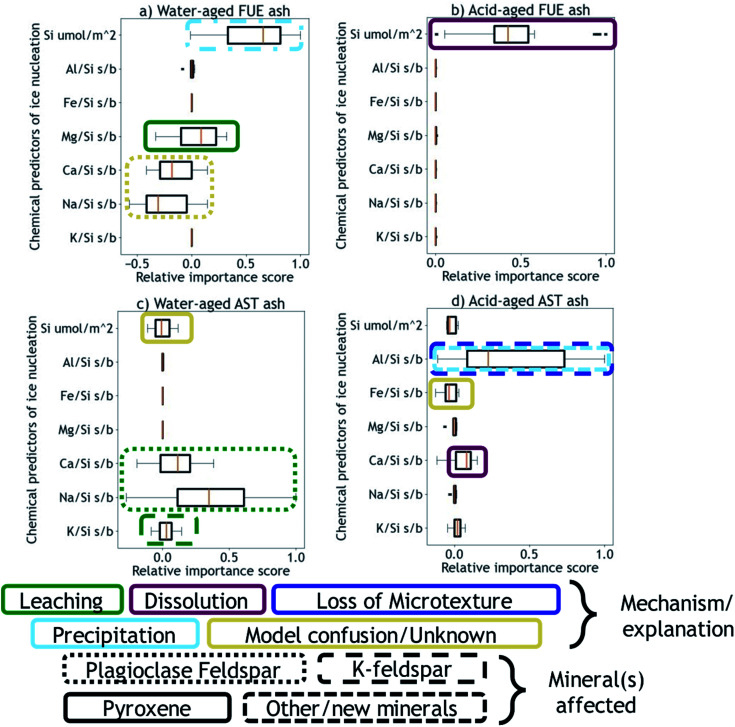
Permutation importances of mean element signatures to the predictive model of INA for FUE ash aged in (a) H_2_O (water-aged) or (b) pH 1.75 H_2_SO_4_ (acid-aged), and AST ash aged in (c) H_2_O (water-aged) or (d) pH 1.75 H_2_SO_4_ (acid-aged). All values are scaled such that 1.0 represents the maximum positive importance in each dataset. The proposed reaction mechanisms and the mineral(s) affected are indicated by the annotations using the box color and box line style, respectively.

Next, using the Python machine learning library scikit-learn^89^ a nonlinear Support Vector Machine regressor model^90,91^ was built and tested between the mean dissolved element signatures and the INA difference vectors corresponding to each aging experiment of a given duration. Permutation importances^92^ were calculated to quantify how important each element signature is for predicting the changes in INA observed for each ash/aging solution pair, shown in Fig. 3. A positive permutation importance means that the model uses some aspect of that element signature to predict changes in INA. Negative values indicate the model is improved by randomization, which could mean that the model incorrectly expects them to be important based on other data. A permutation importance of zero does not necessarily imply that a given element signature is uncorrelated with ice nucleation descriptors – it simply means the model does not use that signature to predict INA. Note that permutation importances are more sensitive than correlation coefficients, such that a single mineral phase contributing to an overall element signature could be the cause of a positive permutation importance in that element. Further discussion, testing, and validation of this method can be found in the ESI.† The correlation coefficients and permutation importances are quantitative metrics relating dissolved element signatures to changes in INA, and will be used to inform the following discussion of physical and chemical mechanisms by which the INA of FUE and AST ash might be altered in aqueous environments.

### 4.2 Leaching of mineral surfaces

We propose that formation of silica-like surface layers during aqueous leaching^62,65,67^ of volcanic ash is one of the main drivers of chemical aging deactivating INA in these ash samples. At 10 min of aging in H_2_O, the immediate drop in *n*_m_ of FUE ash across the full range of temperatures and at colder temperatures in the freezing spectrum of AST ash (<−17 °C; Fig. 2a and c) is inferred to result from the development of a cation-depleted and Si(/Al)-enriched surface on pyroxene and feldspar minerals that would otherwise be more ice-active. This hypothesis is supported by high relative concentrations of alkali and alkaline earth metals in solution at early H_2_O aging time points (Table S2†) and by the positive correlation coefficients between these elements and ice nucleation descriptors for both ash samples (Fig. S2 and S4†). In addition, the positive permutation importance of Mg for FUE ash (Fig. 3a) may reflect the importance of leaching of Mg-rich pyroxene^93,94^ to changes in INA, while the negative permutation importances of Ca and Na may indicate that leaching of Na/Ca-feldspar (the dominant phase in FUE ash) is not a significant contributor to the aged ash's ice nucleation behavior. Cations are also likely released by leaching of glass surfaces on FUE and AST ash, but glass is poorly ice-active so its alteration is not expected to drive the changes in INA observed here.^16,43^ The freezing spectra of both FUE and AST ash show reduced activity after aging 10 min of aging in H_2_SO_4_, but little change after longer aging durations (Fig. 2b and d), so leaching of pyroxene and/or feldspar surfaces under acidic conditions may similarly impair ice nucleation on short time scales in those experiments.

Formation of a cation-depleted and Si(/Al)-enriched surface layer is hypothesized to lower INA by reducing its capacity for further uptake of H^+^/H_3_O^+^ in H_2_O, since surface protonation has been proposed to be a key step in ice nucleation by silicate materials containing exchangeable cations.^24^ Moreover, if aging entails dissolution–reprecipitation alongside or instead of leaching at the mineral–solution interface (Yuan *et al.* and references therein^69^), the resulting Si(/Al)-enriched layer is likely to be more amorphous than the original mineral surface,^67,69^ which could also contribute to lowering INA by reducing the lattice match of the surface sites with ice polymorphs.^16,24,37,41,43,95^

At longer time scales in H_2_O, leaching is expected to slow as cations diffuse from progressively greater depths within the solid materials to exchange with H^+^/H_3_O^+^ in solution (Oelkers and references therein^61^), although the rate of leaching of different glass and mineral surfaces on FUE and AST ash will vary. For example, cations are likely released more readily from pyroxene and Na/Ca-feldspar than from K-feldspar, based on the Goldich dissolution series (Fig. 1). This disparity in leaching rates of different minerals may explain the INA trends of AST ash aged in H_2_O, where activity consistent with pyroxene and/or Na/Ca-feldspar at colder temperatures (<−17 °C) is lost after 10 min, while activity consistent with K-feldspar at warmer temperatures (>−17 °C) persists for longer, until it too is steadily suppressed. The positive permutation importances of Na, Ca, and K for AST ash aged in H_2_O (Fig. 3c) conform to this hypothesis. Eventually, the INA of the ash may be impaired by leaching of all mineral surfaces in H_2_O, although this effect may be convoluted by precipitation of secondary phases of variable INA (see Section 4.3). Others have found the INA of microcline (a K-feldspar) to be unaffected or only slightly reduced (freezing temperature lowered by up to 2 °C) following suspension in H_2_O from one week to several months.^19,21,96^ The samples tested in those studies might be more stable than the K-feldspar in AST ash, and/or less ice-active secondary phases might contribute to lowering the INA of AST ash (see Section 4.3).

### 4.3 Precipitation of secondary phases from solution

As dissolved element concentrations increase in the aging solutions, precipitation of secondary phases becomes possible in the free solution and/or on nucleation sites on ash surfaces, especially in the experiments in H_2_O where pH is expected to be near-neutral. This phenomenon has been reported previously to influence the INA of quartz and simple silicate materials,^24,25^ but has not previously been reported in the context of volcanic ash. Table 2 lists saturation indices associated with the aging of FUE and AST ash in H_2_O and H_2_SO_4_, calculated from measured dissolved element concentrations using the geochemical modeling software PHREEQC.^97^

**Table 2:** Saturation indices of selected minerals predicted by PHREEQC from dissolved element concentrations measured in the H_2_O and H_2_SO_4_ aging solutions for FUE and AST ash. Saturation index is defined as the log_10_ of the ion activity product divided by the solubility product of a mineral phase. A positive value indicates that a mineral phase is supersaturated and may precipitate, while a negative value indicates that the phase is undersaturated and its component elements are likely to remain in solution

Ash sample	Aging solution/duration	Saturation index of selected minerals					
		Montmorillonite	Kaolinite	Gibbsite	K-mica	Crystalline SiO_2_^a^	Al-oxyhydroxide^b^
FUE	H_2_O/10 min	−0.1	3.8	2.6	—	−1.1/−1.5/−1.6	2.1/3.8
	H_2_O/1 h	0.4	3.9	2.4	—	−0.9/−1.3/−1.3	2.0/3.7
	H_2_O/4 h	1.7	4.8	2.7	—	−0.7/−1.1/−1.1	2.2/3.9
	H_2_O/24 h	2.9	5.6	2.9	—	−0.5/−0.9/−0.9	2.4/4.1
	H_2_O/120 h	4.2	6.5	3.1	—	−0.3/−0.7/−0.7	2.6/4.4
AST	H_2_O/10 min	1.6	4.7	2.6	7.9	−0.6/−1.0/−1.1	2.1/3.8
	H_2_O/1 h	2.7	5.4	2.7	9.0	−0.4/−0.8/−0.9	2.2/4.0
	H_2_O/4 h	3.7	6.0	2.8	10.0	−0.3/−0.6/−0.7	2.4/4.1
	H_2_O/24 h	5.4	7.2	3.2	11.9	0.0/−0.4/−0.4	2.7/4.4
	H_2_O/120 h	5.6	7.3	3.1	12.0	0.1/−0.3/−0.4	2.7/4.4
FUE	H_2_SO_4_/10 min	−16.4	−11.1	−6.7	−21.1	0.7/0.3/0.3	−7.1/−5.4
	H_2_SO_4_/1 h	−14.6	−9.9	−6.4	−19.4	1.1/0.7/0.6	−6.9/−5.2
	H_2_SO_4_/4 h	−13.1	−8.9	−6.2	−17.9	1.3/0.9/0.9	−6.7/−5.0
	H_2_SO_4_/24 h	−12.0	−8.3	−6.1	−16.9	1.6/1.2/1.1	−6.6/−4.9
	H_2_SO_4_/120 h	−11.7	−8.1	−6.1	−16.6	1.6/1.2/1.2	−6.5/−4.8
AST	H_2_SO_4_/10 min	−18.3	−12.1	−6.6	−21.2	0.2/−0.2/−0.3	−7.1/−5.4
	H_2_SO_4_/1 h	−17.4	−11.5	−6.4	−20.2	0.3/−0.1/−0.1	−6.9/−5.2
	H_2_SO_4_/4 h	−16.8	−11.1	−6.3	−19.6	0.4/0.0/0.0	−6.8/−5.1
	H_2_SO_4_/24 h	−15.8	−10.4	−6.2	−18.5	0.6/0.2/0.2	−6.7/−5.0
	H_2_SO_4_/120 h	−14.6	−9.6	−6.1	−17.4	0.8/0.4/0.4	−6.5/−4.8

a Quartz/cristobalite/chalcedony.

b Boehmite/diaspore.

In aging experiments of both FUE and AST ash in H_2_O, secondary mineral phases such as montmorillonite, kaolinite, and gibbsite are found to be supersaturated in solution, indicating that these phases could have precipitated and been retained with the filtered ash particles after aging. All three of these minerals have previously been observed to nucleate ice,^26,28,41^ such that precipitation of ice-active phases may contribute to the observed recovery of INA of FUE ash from the leached state at aging durations longer than 10 min in H_2_O. The strongly positive permutation importance of Si for FUE ash aged in H_2_O (Fig. 3a) could reflect Si concentration representing the total quantity of elements released from the ash and contributing to precipitation of secondary phases, consistent with the steady increase in saturation indices over time. The non-monotonic change in INA of FUE ash aged in H_2_O overall might thus reflect competing influences of leaching and precipitation, but additional experiments and analyses would be needed to unravel their individual contributions. No recovery in INA is observed for AST ash aged in H_2_O over time, even though montmorillonite, gibbsite, and kaolinite are also supersaturated in the aging solutions; this conforms with these minerals being much less ice-active than the K-feldspar largely driving the INA of AST ash.

In addition to potentially introducing new ice-active sites, secondary mineral phases are often more disordered than the primary mineral phases from which they originate or are naturally weakly ice-active, such as K-mica which could precipitate onto AST ash aged in H_2_O. If such poorly crystalline or weakly ice-active secondary phases nucleate on or adsorb onto the aged ash surface,^98^ they could impair INA by covering more active ice nucleation sites. Relatedly, a decrease in INA of quartz observed over five days in H_2_O has been attributed to the adsorption of supersaturated silicic acid onto the quartz, covering a fraction of the ice-active sites on the quartz surface.^27^ This effect is expected to be time-dependent, with longer leaching durations leading to greater concentrations of secondary phases and hence more masking of original ice-active surfaces. As in FUE ash aged in H_2_O, a combination of leaching and precipitation might contribute to the overall changes in INA observed for AST ash aged in H_2_O. For example, the drop in *n*_m_ at temperatures <−17 °C after 10 min of aging may be due to fast leaching of pyroxene and/or Na/Ca-feldspar, with a time-dependent decrease in *n*_m_ thereafter (particularly at warmer temperatures) reflecting further leaching and/or masking of mineral surfaces by poorly ice-active aluminosilicate or Al-oxyhydroxide precipitates (Fig. 2c).

In aging experiments of both FUE and AST ash in H_2_SO_4_, most minerals are undersaturated except for some crystalline SiO_2_ phases, which are predicted to be slightly supersaturated. These phases could have precipitated and contributed to the loss of ice-active sites by nucleation or adsorption on ash surfaces aged in H_2_SO_4_, particularly in acid-aged FUE ash, where the positive permutation importance of Si (Fig. 3b) and positive correlation between Si and ice nucleation descriptors (Fig. S3†) indicate that a process affecting dissolved Si concentrations is the main driver of changes in INA. In acid-aged AST ash, the influence of precipitation of SiO_2_ phases on INA is expected to be minor compared to that of dissolution of strongly ice-active components (see Section 4.4), as these phases are barely saturated on the time scales of this experiment. For both ash samples, precipitation of Al-bearing secondary minerals is likely inhibited by low pH and by complexation between aqueous SO_4_^2−^ and Al in the experiments in H_2_SO_4(aq)_.^99^

### 4.4 Dissolution of mineral surfaces

We hypothesize that variable dissolution of silicate surfaces also underlies the effects of chemical aging on INA, especially in acidic solution. Increasing dissolved Si concentrations in leachates of FUE and AST ash with increasing aging duration support dissolution of the ash over time, and higher dissolved Si concentrations from ash samples aged in H_2_SO_4_ compared to those aged in H_2_O are consistent with greater dissolution at lower pH.^68,100^ Acid-mediated breakdown of the silicate network is also facilitated by aqueous complexation of SO_4_^2−^ and Al.^99^ The increase in SSA_BET_ over time of FUE and AST ash aged in H_2_SO_4_ also supports extensive dissolution of these silicate materials, as a reduction in overall particle size and selective dissolution at etch pits and other surface textures^69,101–103^ could both contribute to increasing the SSA_BET_ of the ash samples.

After 10 min of aging in H_2_SO_4_, the freezing spectra of FUE ash shows a slight decrease in INA relative to the non-aged ash (Fig. 2b), potentially due to accelerated leaching of pyroxene surfaces at low pH. This deactivation is followed by a partial recovery of *n*_m_ after 1 h and then full recovery to the active site densities observed in the non-aged ash from 4 to 24 h, in parallel with the increase in SSA_BET_. Similarly, the INA at <−17 °C of AST ash exposed to H_2_SO_4_ is largely preserved from 10 min to 120 h of aging (Fig. 2d), as SSA_BET_ increases.

As noted previously, pyroxene is inferred to drive ice nucleation by FUE ash at all temperatures and in part by AST ash at temperatures <−17 °C, so the observed preservation of INA in most acid-aged ash samples presumably relates to alteration of pyroxene in H_2_SO_4_. Zakaznova-Herzog *et al.* found that pyroxene alteration at low pH involves formation of a leached monolayer which is rapidly broken down, liberating Si to solution.^104^ Other studies have also observed very thin cation-depleted leached layers under acidic conditions which may not change crystal structure when cations are leached prior to Si release.^61,103^ Similarly, thin leached layers have been reported for some feldspars, for example on the Ca-rich endmember of Na/Ca-feldspar (anorthite), where an Al : Si ratio of one results in near-congruent dissolution.^59,105–108^ We hypothesize that the relatively stable INA maintained by the acid-aged FUE ash and AST ash (<−17 °C) reflects a quasi-steady-state density of ice-active sites across the ash surfaces, with some sites lost and others created during simultaneous leaching and dissolution in H_2_SO_4_. Dissolution of thin leached layers on pyroxene and potentially Na/Ca-feldspar might expose underlying surfaces characterized by a higher density of ice-active sites. Alternatively or additionally, surface –OH or –OH_2_^+^ groups that can enhance INA^29,42,109^ might be generated as Al–O and Si–O bonds are cleaved by H^+^ at the mineral–solution interface.^24,67,110^

The positive permutation importance of Si for acid-aged FUE ash (Fig. 3b) could indicate that Si release, particularly from the dissolution of pyroxene in H_2_SO_4_, is key for predicting the stability of this samples' INA. Additionally, Si and Mg are correlated with ice nucleation descriptors in acid-aged FUE ash (Fig. S3†) and Si is correlated with ice nucleation descriptors in acid-aged AST ash (Fig. S5†), which may correspond to small changes in the position of the quasi-equilibrium state of ice-active sites as the aging solution becomes more saturated. The small or zero permutation importances of Na and K for both ash samples aged in H_2_SO_4_ and the anticorrelations of these metals with ice nucleation descriptors may indicate that leaching of feldspars and other Na- and K-containing components is unimportant for predicting changes in INA for these ash samples, highlighting the role of other transformations in altering the INA of AST ash. The slightly positive permutation importance of Ca for acid-aged AST ash may reflect the contribution of more Ca-rich pyroxene dissolution in H_2_SO_4_ to INA trends (<−17 °C) for this sample, where Ca serves as the element descriptor of the leaching/dissolution quasi-equilibrium of pyroxene, instead of Si as in acid-aged FUE ash.

In contrast to the INA associated with pyroxene, the INA at >−17 °C of AST ash exposed to H_2_SO_4_ is strongly deactivated at 10 min and continues to decrease up to 120 h of aging, presumably reflecting a loss of ice nucleation active sites on K-feldspar at the ash surface. Like many aluminosilicate minerals, leaching and dissolution of K-feldspar is accelerated at low pH relative to neutral conditions.^68,100^ While K-feldspar is one of the most stable minerals in volcanic ash (Fig. 1), H_2_SO_4_ may selectively destroy high energy patches and/or perthitic microtextures thought to give rise to this mineral's exceptional INA.^22,33,34,42^ The high INA of AST ash has been attributed to similar features in its K-feldspar phases,^16,111^ so removal of these features could explain the drop in INA at >−17 °C of AST ash exposed to H_2_SO_4_ here. Numerous studies show that microtextures and dislocations dissolve preferentially or even control the dissolution rate of K-feldspar (Wilson and references therein^68^). Lastly, complexation of SO_4_^2−^ and Al on K-feldspar surfaces might also impair INA by blocking ice-active sites and/or removing –OH groups, as has been suggested for microcline immersed in various SO_4_^2−^-containing solutions, with no such impairment observed for Na/Ca-feldspars.^19,24,99^ Both mechanisms of INA impairment of K-feldspar in H_2_SO_4_ may be reflected in the high permutation importance of Al for the acid-aged AST ash (Fig. 3d), where the distinct importance of Al rather than Si might point to the dominance of K-feldspar dissolution over pyroxene dissolution in predicting the overall changes in INA. There is also a direct correlation between Al and ice nucleation descriptors (Fig. S5†), as would be expected if dissolution of Al-containing phases such as feldspar was driving changes in INA and/or if Al/SO_4_^2−^ surface complexes are impairing the INA of the acid-aged AST ash. These correlations vary by ice nucleation descriptor, and since different descriptors represent different aspects of the curvature of the freezing spectrum, this is consistent with the change in shape of the spectrum observed in the K-feldspar regime of AST ash aged in H_2_SO_4_, further supporting the importance of Al.

The aging effects observed here contrast with those of previous aging experiments on volcanic ash, but the disparities are likely caused by differences in processing conditions. Maters *et al.*^43^ showed that exposure to H_2_O_(g)_ on its own or mixed with HCl_(g)_ reduced the INA of Astroni ash, while exposure to H_2_O_(g)_ mixed with SO_2(g)_ could enhance INA, but these high temperature (up to 800 °C) gas–ash interaction experiments are not comparable to aqueous aging at ambient temperature. Experiments performed by Jahn^56^ showed a reduction in Fuego ash INA after five days immersed in 0.01 M H_2_SO_4(aq)_ and no change in INA upon immersion in H_2_O_(l)_, but the slow drying procedure used may have altered the pH and saturation conditions within the aging solution, thereby influencing the relevant mechanisms of chemical processing.

Notably, previous experiments on mineral dust and/or individual mineral phases present in the ash samples studied here are broadly consistent with our findings. Arizona Test Dust (ATD, which contains feldspars) aged in H_2_O_(l)_ and H_2_SO_4(aq)_ by Perkins *et al.* shows similar sensitivities in its freezing spectrum depending on aging solution: ice nucleation sites active at colder temperatures (<−12 °C) are degraded in H_2_O_(l)_ but largely preserved in H_2_SO_4(aq)_ (as for FUE ash and partly for AST ash) and the most active ice nucleation sites associated with K-feldspar (>−12 °C) are largely preserved in H_2_O_(l)_ but degraded in H_2_SO_4(aq)_ (as for AST ash).^50^ Sullivan *et al.* also reported preservation of immersion-mode INA in aerosolized ATD exposed to HNO_3_ vapor,^54^ and Kulkarni *et al.* reported a similar preservation in immersion-mode INA in ATD, illite, montmorillonite, K-feldspar, and quartz exposed to H_2_SO_4_ vapor.^112^ In other cases, irreversible losses of immersion-mode INA in ATD,^53,113^ kaolinite,^114^ and feldspars^115^ were observed when exposed to H_2_SO_4_ vapor, although the presence of H_2_O_(g)_ and use of heat to produce the H_2_SO_4_ vapor may play an important role in this process. Similarly, Yun *et al.* recently showed a pH-sensitive deactivation of K-feldspar INA when exposed to H_2_SO_4(aq)_, HNO_3(aq)_, HCl_(aq)_, and aqueous organic acids.^116^ Finally, Losey *et al.* showed that formation and precipitation of gypsum (CaSO_4_·2H_2_O) on fly ash exposed to H_2_SO_4(aq)_ can reduce the INA of the original minerals, while aging without the possibility of gypsum precipitation slightly enhances the INA of the fly ash samples.^51^ While these observations corroborate the explanations proposed for the changes in the INA of volcanic ash here, interplay between leaching, precipitation, dissolution, and possibly other mechanisms affecting the INA of the various materials hinders direct comparison to the observations for FUE and AST ash.

## 5 Conclusions and implications

**Fig. 4:**
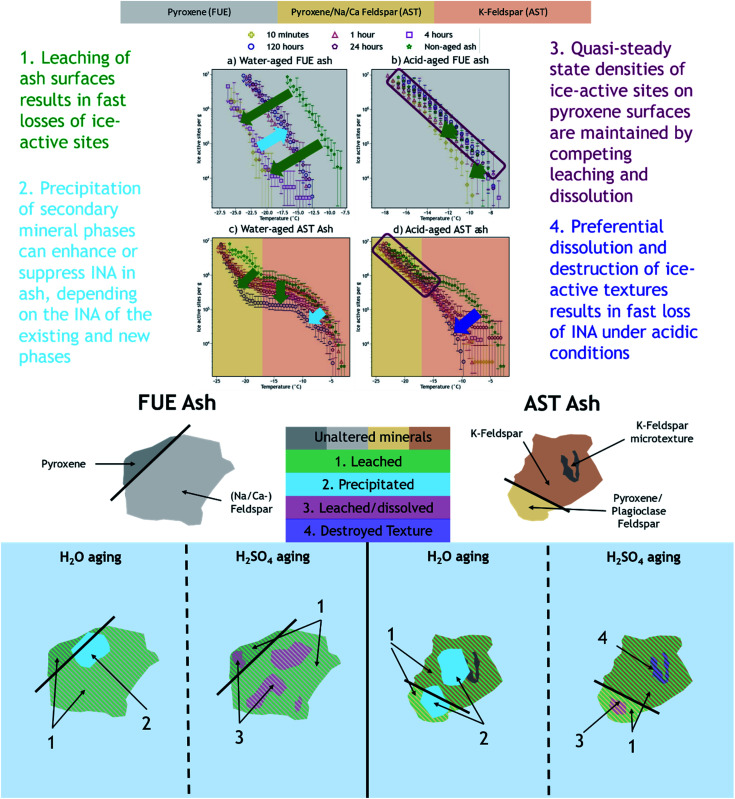
Attribution of ice nucleation spectral features to specific mineral phases and summary of proposed mechanisms by which chemical aging impacts the INA of FUE and AST ash. Note the full composition of each ash is not represented because not all minerals are expected to contribute to the observed ash INA.

We have shown that the effects of aqueous chemical aging in H_2_O or H_2_SO_4_ on the INA of two volcanic ash samples depend on the aging duration, ash mineralogy, and ice nucleation temperature regime. We infer that the INA of FUE and AST ash exposed to H_2_O was reduced on minute to hour time scales in the temperature regimes dominated by pyroxene and perhaps Na/Ca-feldspar (at all temperatures for FUE ash and <−17 °C for AST ash) due to formation of a Si(/Al)-rich layer on these mineral surfaces, while the temperature regime dominated by the more chemically stable K-feldspar (>−17 °C in AST ash) was less affected. Further enhancements and reductions with aging duration in the INA of FUE and AST ash, respectively, may be the result of precipitation of secondary phases of variable INA from the H_2_O aging solution. When exposed to H_2_SO_4_, in the temperature regime where ice nucleation may be controlled by pyroxene or Na/Ca-feldspar, a quasi-steady state INA is observed. This may reflect parallel leaching and dissolution of ash resulting in a competition between loss and exposure of new ice-active sites on mineral surfaces. Finally, the fast and dramatic loss of INA of AST ash in the temperature regime attributed to K-feldspar is hypothesized to result from fast dissolution of high-energy surface microtextures that have been shown to account for most of the INA of some types of K-feldspars. Fig. 4 provides a summary of the proposed aging effects reflected in the INA trends of FUE and AST ash aged in H_2_O or H_2_SO_4_.

While previous studies on individual silicate minerals or mineral dust proxies often show small losses of INA in acid and sometimes report changes in INA from suspension in H_2_O,^24,25,51,53–55,96,112,115^ the 10 °C difference between the freezing spectra of the non-aged and water-aged FUE ash is striking and the non-monotonicity of its INA with aging duration in H_2_O is unprecedented. Clearly, more research is required to understand the complexities of the effects of chemical alteration on INA, especially for less-studied minerals such as pyroxenes and for multicomponent materials like volcanic ash, natural mineral dust, and biomass-burning aerosol^117,118^ that are present in the atmosphere.

In addition to aqueous chemical aging, exposure to gaseous acids, oxidants, and other pollutants can alter the INA of silicate materials,^31,43,119^ both in the wider atmosphere and – in the case of volcanic ash – at high temperatures in the eruption plume. When these processes occur in parallel or in series, they may change the INA of the airborne particles in unexpected ways, leading to additional uncertainty about their capacity for ice nucleation during atmospheric transport. For example, a potential enhancement of INA due to formation of ice-active anhydrite (CaSO_4_) on ash surfaces by reaction with gaseous SO_2_ in the hot eruption plume^43^ may be offset by a loss of INA during subsequent ash leaching and dissolution in aqueous H_2_SO_4_ and H_2_O in the cooling plume and wider atmosphere. Laboratory studies will play a key role in unravelling the complexity of such competing effects. Further investigations are needed on the influence of various factors (*e.g.*, other mineralogies, other aging procedures, particle size, solution temperature and pH, presence of other solutes) that could affect the impact of aqueous aging on the INA of silicate materials.^81,116,120,121^ Also, the influence of gas-phase chemical processing on the INA of silicate aerosol *via* flow tube or chamber experiments is worthy of future study. Findings from such studies will have implications for understanding the INA of both primary volcanic ash emissions and resuspended ash material which has undergone further physicochemical alteration following deposition, including by leaching, dissolution and precipitation upon contact with aqueous solution (*e.g.*, rainwater) in the environment.

Elucidating the influence on INA of chemical and physical variables individually and deconvoluting the molecular-scale mechanisms of aging and atmospheric pathways of solid–gas/liquid exposure will be important next steps towards a deeper understanding of ice nucleation by airborne particles. Many of these variables should be studied under laboratory conditions to control potential convoluting factors, and applying surface sensitive physicochemical analyses (*e.g.*, Yun *et al.*^121^) to aged materials will be particularly helpful for studying the mechanisms of alteration of INA. Fieldwork and modelling studies will also be necessary to constrain the real-world impacts of aging *via* different processes on atmospheric INP concentrations and activity. Without further research, it is difficult to predict on the basis of our findings how the INA of volcanic ash will change as it ages in the environment, but understanding the geochemical mechanisms underlying aging is necessary for a more complete picture of heterogeneous ice nucleation and its evolution in the atmosphere.

## Data availability

The dataset associated with this article is available in the Research Data Leeds Repository at https://doi.org/10.5518/1079.

## Author contributions

Conceptualization: all co authors; investigation & methodology: WDF, ECM, RGM and MPA; funding acquisition, resources & supervision: ECM, RCS and BJM; formal analysis: WDF; writing – original draft & visualization: WDF and ECM; writing – review & editing: WDF, ECM, MPA, LGJ, RCS and BJM.

## Conflicts of interest

There are no conflicts of interest to declare.

## Supplementary Material

EA-002-D1EA00071C-s001
