# Supplementary material for: Volcanic ash ice nucleation activity is variably reduced by aging in water and sulfuric acid: the effects of leaching, dissolution, and precipitation
Source: Environ Sci Atmos. 2021 Dec 22;2(1):85–99. doi: 10.1039/d1ea00071c (PMC8772422; doi:10.1039/d1ea00071c)
Supplement: EA-002-D1EA00071C-s001 [file EA-002-D1EA00071C-s001.pdf]

Electronic Supplementary Material (ESI) for Environmental Science: Atmospheres.
This journal is © The Royal Society of Chemistry 2021

Supplementary Information

Bulk chemical composition analysis of ash samples

The bulk chemical composition of FUE and AST ash (Table S1), expressed as oxide wt.%, was determined by X-ray fluorescence analysis by Stanley Mertzman (Department of Earth & Environment, Franklin and Marshall College, USA) and Nora Groschopf (Institute of Geosciences, Johannes Gutenberg University Mainz, Germany), respectively.

Table S1. Bulk chemical composition as oxide wt.%, normalized to 100 wt.% (excluding loss on ignition), and specific surface area (SSA_{BET}) of the ash samples studied.

Sample	SiO_2	Al_2O_3	Fe_2O_3	MgO	CaO	Na_2O	K_2O	TiO_2	MnO	SSA_{BET} (g/m^2)
FUE	54.2	20.9	7.6	2.7	8.9	3.9	0.9	0.8	0.1	1.5
AST	59.5	18.9	4.2	0.9	3.2	4.0	8.6	0.5	0.1	3.2

Background subtraction and statistical treatment of ice nucleation data

The sample data were first binned by temperature with a 0.5 °C bin width allowing the number of droplets that freeze in each bin to be resampled 100 times from a Poisson distribution. Each resampling across all temperature bins is then treated as a simulated experiment with its own freezing spectrum. The 2.5th and 97.5th quantiles of these simulated spectra experiments are then used to approximate the 95% confidence interval around each binned datapoint. Pure water background freezing spectra are subtracted from each sample through the differential spectrum with 2-sided propagation of error to account for both sets of confidence intervals. The differential spectra are then numerically re-integrated with propagation of error to produce the background subtracted INA spectra. Finally, data from experiments examining the same sample at different suspension concentrations were combined by averaging the differential n_s spectra over each temperature bin with propagation of error. The cumulative n_s spectra were then recalculated by numerical integration as above.

Regression model between dissolved element signatures and descriptors of ice nucleation activity

An epsilon-nonlinear support vector regression model with a radial basis function kernel, a degree of three, a regularization parameter of six and an epsilon of 0.1 was chosen to quantify and predict the relationships between ion signatures and the difference vectors representing changes in INA.[88] Element signatures for all elements except for Si were input as the surface-to-bulk ratio normalized to Si, while Si was input in μmols/m^2 normalized to

the SSA_{BET} of the ash at each aging timepoint (as opposed to the SSA_{BET} of fresh ash) to better represent actual surface-normalized Si dissolution rates. When tested with a leave-one-out cross validation scheme, this model had a test set mean absolute error of 1.69 and a training set error of 1.34 compared to an expected value error of 2.76, all relative to an interquartile range of 1. The free parameters and kernel type were optimized to minimize the test set error. All modelling and testing was performed using the scikit-learn implementations.[88]

Permutation importances on this model were calculated by shuffling each element signature predictor between samples and calculating the resulting loss (or gain) in model training score .[91] Means of the permutation importances are taken to represent the element signature's importance for predicting the overall freezing spectrum, while the spread of each distribution partially represents how much of a given freezing spectrum the element signature predicts. Note that permutation importances are not necessarily related to correlations, and a permutation importance of zero score does not necessarily mean that an ion signature is unrelated to the changes in INA observed. For example, the zero permutation importance of Mg for FUE ash aged in H_2SO_4 does not necessarily mean that alteration of Mg-containing phases is unimportant to INA, since Mg is correlated with Si, which has a positive permutation importance that could support the contribution of such phases to changes in INA. Note that permutation importances are more sensitive than correlation coefficients, such that a single mineral phase contributing to an overall element signal can be the cause of a positive permutation importance in that element.

Table S2. Specific surface area (SSA_{BET}) of FUE and AST ash samples aged in water (-W) or pH 1.75 H_2SO_4 (-A) for different durations, and the corresponding dissolved element concentrations in the aged ash sample leachates reported as mean of three replicates. Dissolved Si is reported normalized to the surface area of the fresh ash, and the remaining elements are reported as a ratio of the element relative to Si in the leachate solution (*s*) over the ratio of the element relative to Si in the non-aged ash bulk (*b*). Numbers in parentheses are standard deviations applied to the last digit with decimal points shown only if the standard deviation crosses the decimal point. Solution to bulk (*s/b*) element ratios greater than one are colored green, while those less than one are colored red.

Sample	SSA_{BET} (g/m^2)	Si ($\mu mol/m^2$)	Al/Si *s/b*	Fe/Si *s/b*	Mg/Si *s/b*	Ca/Si *s/b*	Na/Si *s/b*	K/Si *s/b*	Ti/Si *s/b*	Mn/Si *s/b*
FUE-W-10min	1.6	0.53 (9)	1.5 (3)	-[a]	19.5 (2.2)	54 (7)	34 (4)	-	-	-
FUE-W-1h	1.4	0.91 (1)	0.60 (22)	-	14.5 (5)	35.0 (1.0)	22.3 (1.4)	-	-	-
FUE-W-4h	1.5	1.35 (2)	0.77 (12)	-	10.33 (7)	23.25 (19)	16.9 (5)	-	-	-
FUE-W-24h	1.3	2.17 (9)	0.74 (3)	-	7.0 (3)	14.4 (3)	11.8 (5)	-	-	-
FUE-W-120h	1.5	3.7 (3)	0.7 (3)	-	7 (3)	9.8 (4)	8.25 (9)	-	-	-
FUE-A-10min	3.0	37 (2)	2.37 (4)	3.28 (2)	6.42 (12)	3.98 (6)	1.86 (7)	1.99 (15)	1.06 (3)	4.26 (4)
FUE-A-1h	3.8	81.2 (5)	1.97 (1)	3.23 (9)	7.4 (3)	2.78 (7)	1.15 (3)	0.94 (6)	0.60 (1)	3.82 (14)
FUE-A-4h	4.7	151. (2)	1.91 (2)	2.99 (2)	7.58 (4)	2.21 (1)	0.88 (0)	0.55 (1)	0.33 (0)	3.38 (3)
FUE-A-24h	5.9	245. (2)	1.62 (4)	2.89 (7)	7.84 (20)	2.06 (5)	0.77 (2)	0.40 (1)	0.13 (1)	3.25 (8)
FUE-A-120h	6.6	270 (40)	1.72 (2)	2.64 (3)	5.2 (2.2)	2.13 (6)	0.79 (2)	0.38 (3)	0.02 (0)	3.10 (4)
AST-W-10min	3.3	0.78 (6)	0.54 (14)	-	-	37 (7)	30. (3)	17.3 (1.7)	-	-
AST-W-1h	3.2	1.22 (13)	0.49 (6)	-	-	28.0 (2.2)	21.2 (1.2)	13.7 (8)	-	-
AST-W-4h	3.3	1.85 (11)	0.44 (14)	-	-	21.7 (8)	16.2 (6)	10.7 (6)	-	-
AST-W-24h	3.8	3.29 (23)	0.53 (3)	-	-	12.8 (1.1)	11.3 (3)	7.3 (3)	-	-
AST-W-120h	4.0	3.87 (17)	0.42 (9)	-	-	12.4 (1.0)	10. (7)	6.30 (11)	-	-
AST-A-10min	5.9	4.64 (13)	12.61 (21)	9.66 (5)	12.34 (17)	76.11 (20)	19.03 (14)	24.89 (21)	1.68 (3)	63.3 (4)
AST-A-1h	8.0	6.26 (5)	14.27 (11)	8.51 (8)	10.54 (9)	60.9 (5)	18.78 (16)	19.33 (19)	1.20 (2)	53.4 (3)
AST-A-4h	9.1	8.02 (7)	14.37 (19)	8.08 (6)	10.01 (5)	50.8 (6)	17.16 (6)	16.4 (3)	0.93 (0)	47.73 (24)
AST-A-24h	11.1	12.3 (1)	13.98 (16)	6.70 (4)	8.78 (3)	37.4 (6)	13.94 (22)	12.88 (19)	0.44 (1)	36.6 (8)
AST-A120h	17.6	21.4 (1)	12 (3)	5.32 (4)	6.58 (7)	23.6 (3)	9.30 (10)	8.12 (10)	0.31 (0)	22.86 (11)

LOD (mg/L)	-	0.0349	0.0198	0.120	0.0593	0.0654	0.172	0.0709	0.00172	0.00973
LOQ (mg/L)	-	0.116	0.0659	0.400	0.198	0.218	0.574	0.236	0.00573	0.0324

[a]At least one dissolved element in this ratio was below the LOQ/LOD

Table S3. Collection of point-descriptors of approximate changes in ice nucleation spectra for FUE ash aged in H_2O (FUE-W) or pH 1.75 H_2SO_4 (FUE-A) for different durations.

Sample	Δn_m at -15 °C (orders of magnitude; OOM)	Delta T at 10^4 n_m (°C)	Delta T at 10^6 n_m (°C)	Concavity at -15 °C
FUE-W-10min	(3)[a]	-11	-10	none
FUE-W-1h	1.5	-4	-7	none
FUE-W-4h	3	-11	-10	none->up
FUE-W-24h	1.5	-4	-5	none
FUE-W-120h	1.5	-4	-5.5	none
FUE-A-10min	0.2	-2	-1	none->down
FUE-A-1h	0.1[b]	-1[b]	-0.5[b]	none
FUE-A-4h	0	0	0	none
FUE-A-24h	0	0	0	none
FUE-A-120h	0	0	0	none

[a]Estimated based on the loglinear trend of FUE ash aged in H_2O for 10 minutes. [b]Confidence bands overlap significantly, but the mean values reported are visibly and consistently different.

Table S4. Collection of point-descriptors of approximate changes in ice nucleation spectra for AST ash aged in H_2O (AST-W) or pH 1.75 H_2SO_4 (AST-A) for different durations.

Sample	Δn_m at -10 °C (OOM)	Δn_m at -20 °C (OOM)	ΔT at 10^4 n_m (°C)	ΔT at 10^6 n_m (°C)	Concavity at -10 °C	Concavity at -20 °C
AST-W-10min	0	0.3	0	-3	down	down->up
AST-W-1h	0	0.3	0	-3	down	down->up
AST-W-4h	0.15[a]	0.3	0	-3	down	down->up
AST-W-24h	0.3	0.7	-1	-3	down	down->up
AST-W-120h	0.5	1.4	-2	-3.5	down	down->up
AST-A-10min	1.1	0.3	-5	-2.5	down->none	down->none
AST-A-1h	1.1	0.3	-5	-2.5	down->none	down->none
AST-A-4h	1	0.3	-3	-2.5	down->none	down->none
AST-A-24h	0.9	0	-0	0	down->none	down->none
AST-A-120h	2	0.3	-6.5	-2.5	down-	down->none

[a]Confidence bands overlap significantly, but the mean values reported are visibly and consistently different.

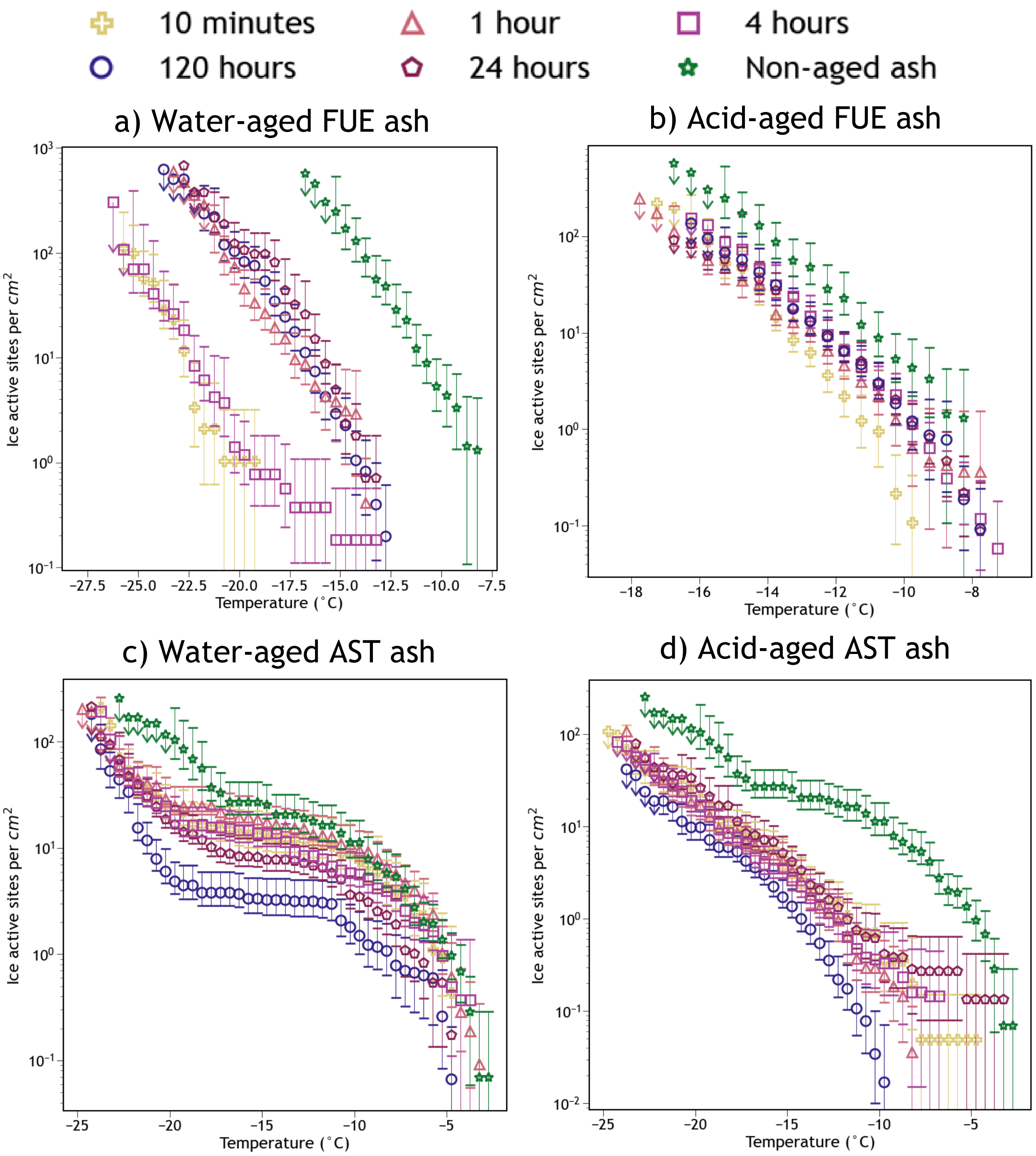

Figure S1: Ice nucleation active site density normalized to surface area (n_s) versus temperature spectra of FUE ash non-aged or aged for different durations in a) H_2O or b) pH 1.75 H_2SO_4, and AST ash non-aged or aged for different durations in c) H_2O or d) pH 1.75 H_2SO_4. The 95% confidence intervals were approximated as the 2.5th and 97.5th quantiles using Monte Carlo simulations based on a Poisson distribution of droplet freezing events. Each spectrum is background-subtracted and is a combination of three experiments on 1, 0.2, and 0.04 wt.% suspensions with error and activity propagated through the differential ice nucleation site density spectra.

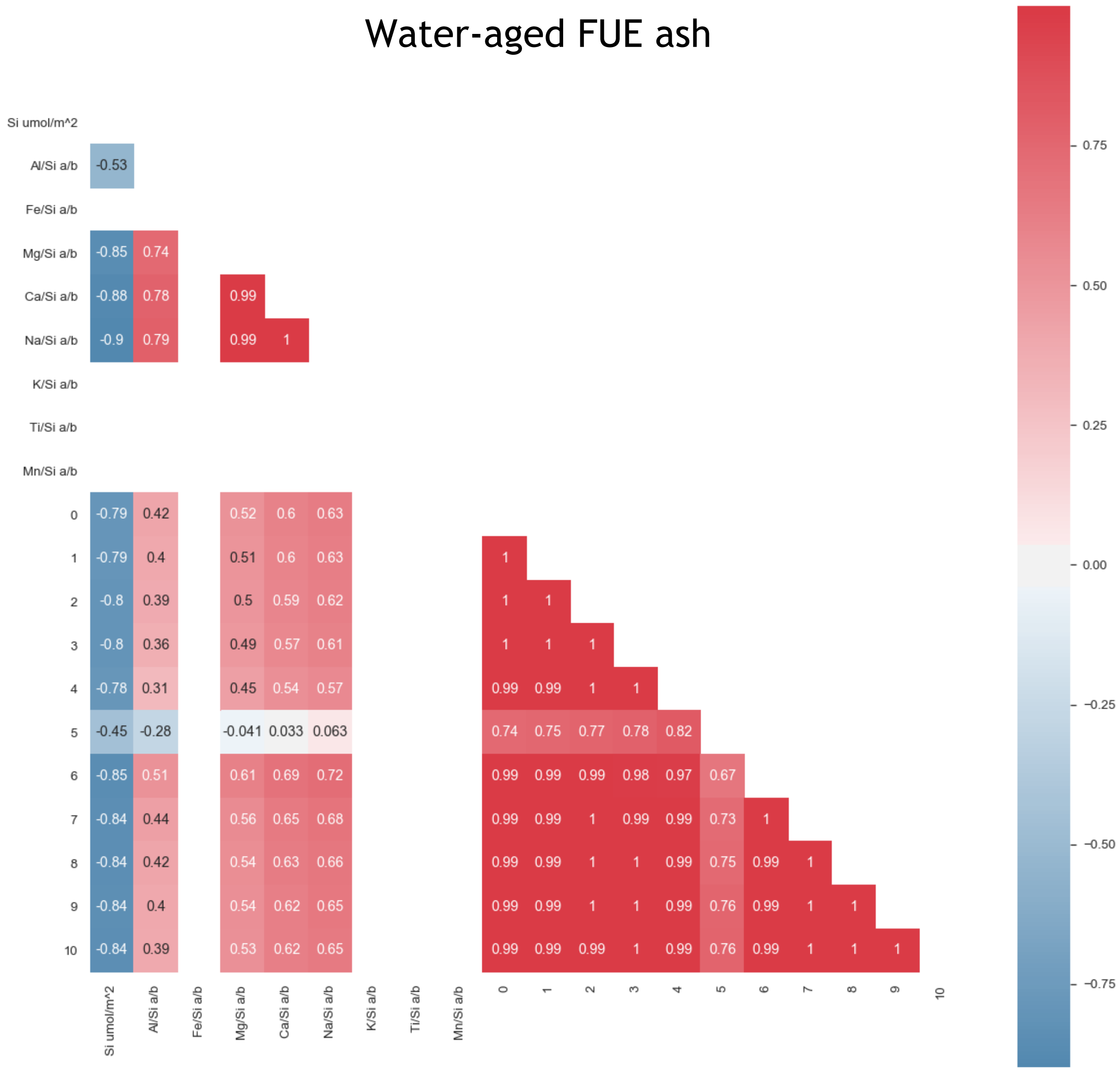

Figure S2: Correlation coefficient heatmap showing elementwise relationships between normalized dissolved element signatures and coefficients of the Chebyshev polynomial representing the quotient of FUE ash aged in H_2O (water-aged) with non-aged FUE ash at each time point. Red indicates a correlation, while blue indicates anticorrelation. Blank spaces appear where these elements were not detected in the aging solution at any timestep.

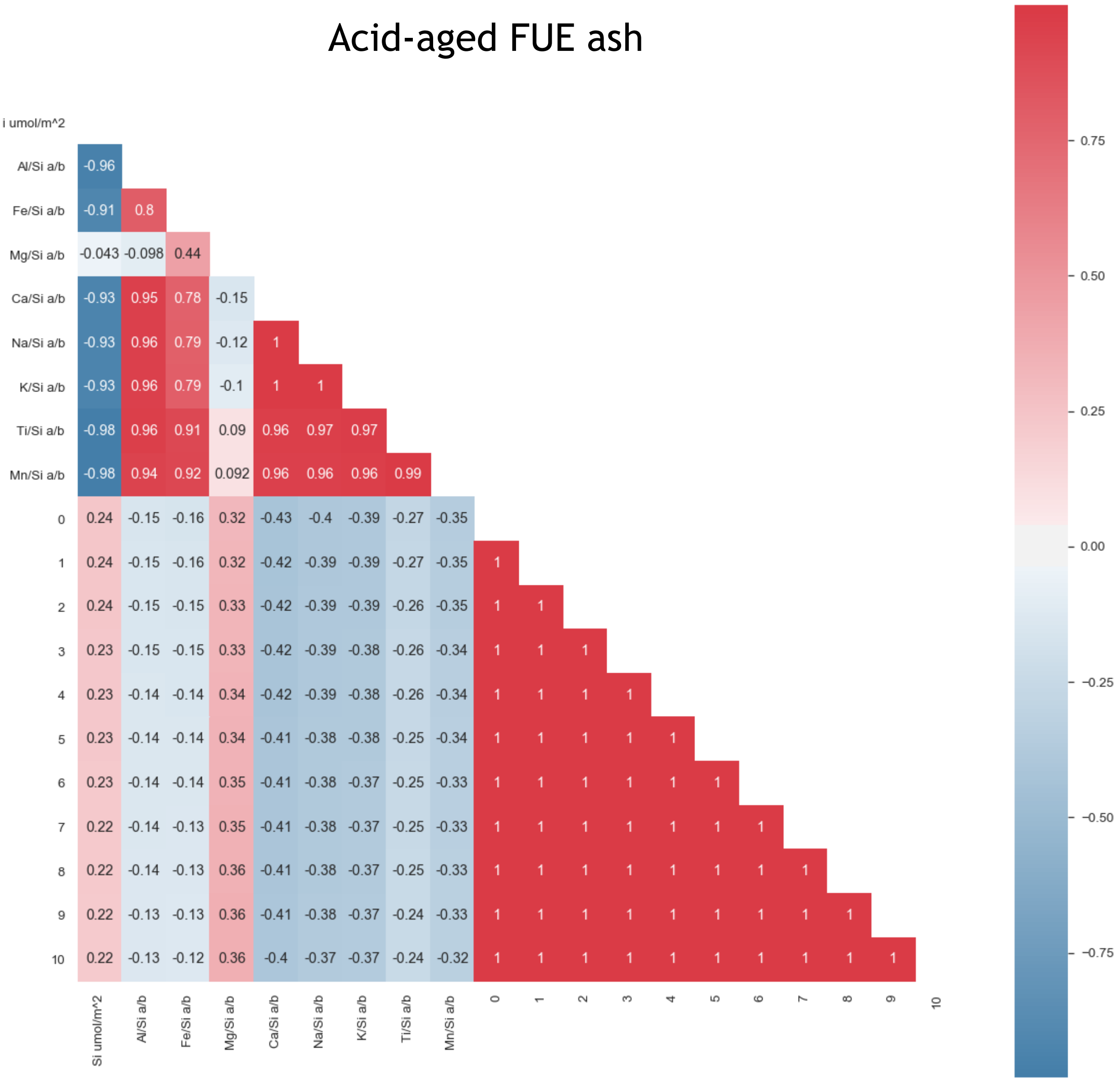

Figure S3: Correlation coefficient heatmap showing elementwise relationships between normalized dissolved element signatures and coefficients of the Chebyshev polynomial representing the quotient of FUE ash aged in pH 1.75 H_2SO_4 (acid-aged) with non-aged FUE ash at each time point. Red indicates a correlation, while blue indicates anticorrelation.

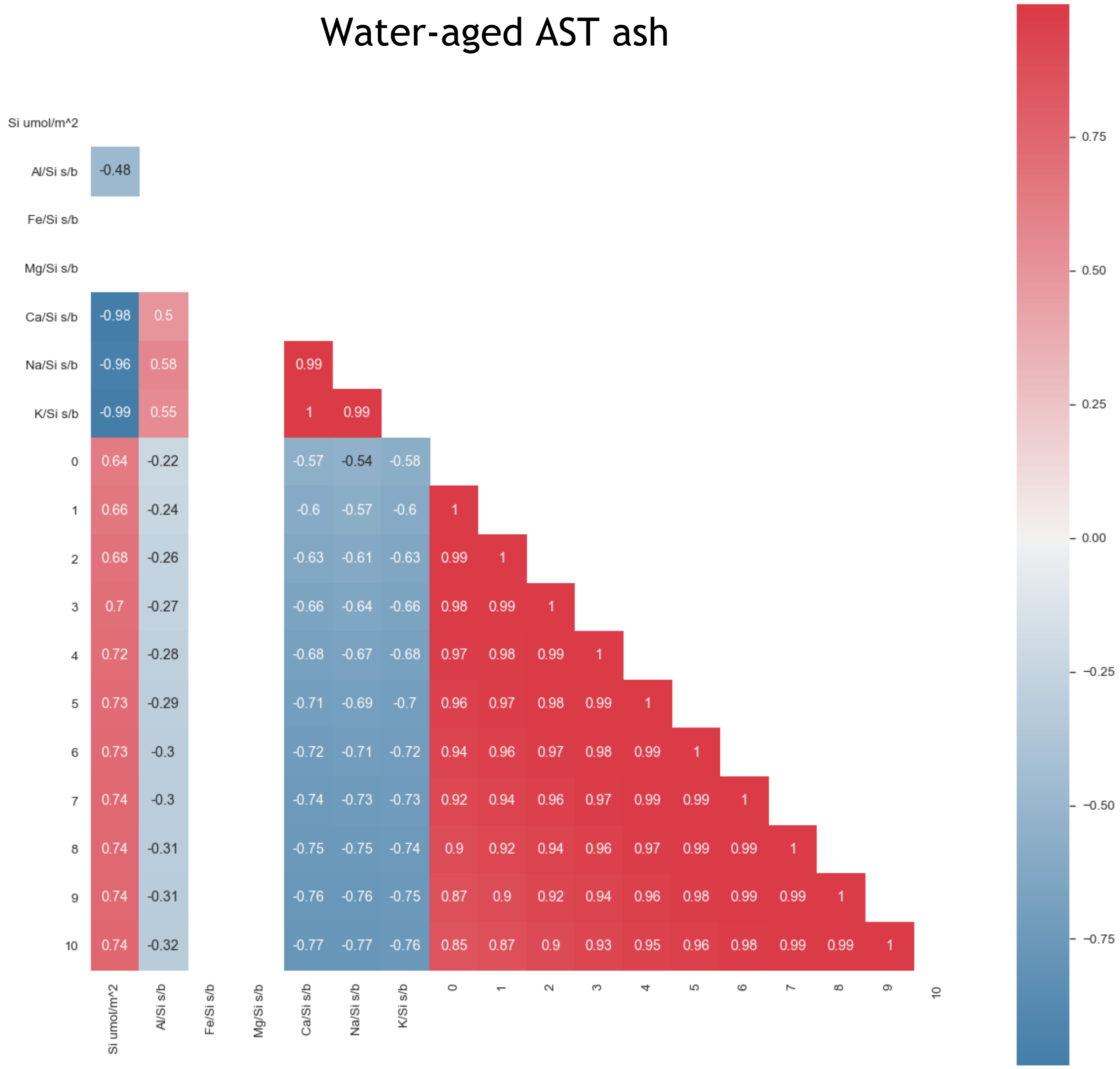

Figure S4: Correlation coefficient heatmap showing elementwise relationships between normalized dissolved element signatures and coefficients of the Chebyshev polynomial representing the quotient of AST ash aged in H_2O (water-aged) with non-aged AST ash at each time point. Red indicates a correlation, while blue indicates anticorrelation. Blank spaces appear where these elements were not detected in the aging solution at any timestep.

Acid-aged AST ash

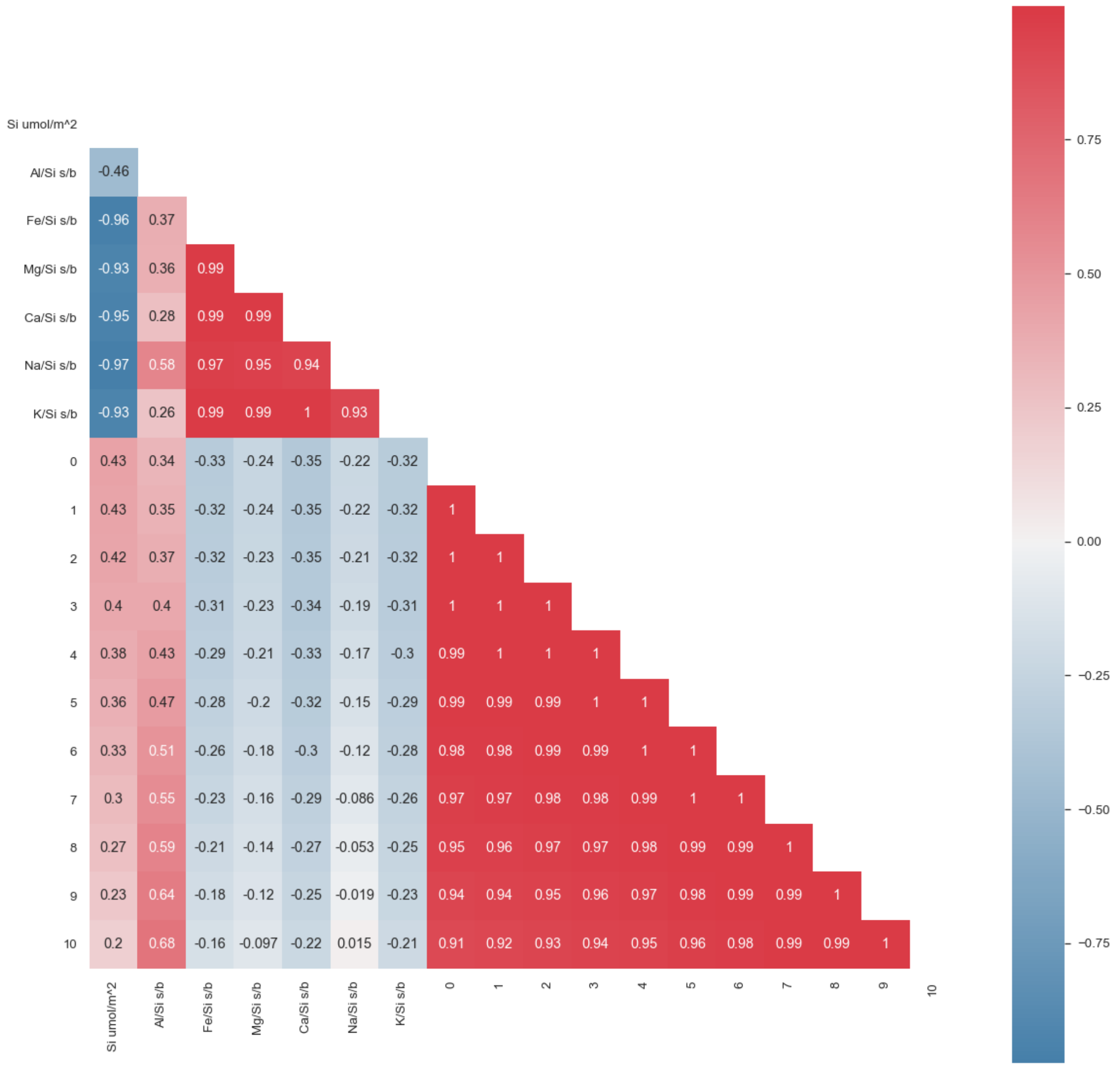

Figure S5: Correlation coefficient heatmap showing elementwise relationships between normalized dissolved element signatures and coefficients of the Chebyshev polynomial representing the quotient of AST ash aged in pH 1.75 H_2SO_4 (acid-aged) with non-aged AST ash at each time point. Red indicates a correlation, while blue indicates anticorrelation.
